# Reply: Clinical outcome and prognostic factors for patients treated within a phase I study: the Royal Marsden Hospital Experience

**DOI:** 10.1038/sj.bjc.6604649

**Published:** 2008-10-14

**Authors:** D Olmos, H-T Arkenau, J E Ang, J S de Bono, I Judson, S B Kaye

**Affiliations:** 1 Drug Development Unit, The Institute of Cancer Research and The Royal Marsden Hospital, Sutton, UK

**Sir,**

We thank the correspondent for his interest in our report. Dr Tirelli has raised important questions regarding the specific subgroup of urological cancer patients included in our previous reported series (Arkenau *et al*, 2008).

Dr Tirelli made the point that 66% of the castration-resistant prostate cancer (CRPC) patients included in the analysis were chemotherapy-naive. In 2004, two phase-III studies (Tannock *et al*, 2004; Petrylak *et al*, 2004) demonstrated an overall survival (OS) benefit approximating a median of 2.5 months for chemotherapy-naive CRPC patients who received docetaxel-based treatment compared to mitoxantrone/prednisone. This led to the approval of this agent in the setting of CRPC in the United Kingdom in June 2006. These circumstances explain why 37 of the 54 CRPC patients included in our analysis were chemotherapy-naive. All the remaining 17 patients received chemotherapy before their phase-I entry (including 10 patients who received prior docetaxel).

The superior outcome of the urological cancer patients subgroup in comparison with the non-urological tumour patients in our series could be attributed to the use of active agents in the former group. In fact, a high proportion of CRPC patients were treated in taxane combination phase-I trials (27 of 37 chemo-naive patients). Furthermore, many patients received abiraterone acetate, a novel CYP17-specific inhibitor, within this analysis or later; this agent has already shown preliminary promising activity (Attard *et al*, 2008). In addition, our urological cancer patients cohort include bladder and renal cancer patients, and many of them were treated within taxane combinations or novel antiangiogenic phase-I trials, respectively.

Although additional retrospective CRPC subgroups analysis comparing outcomes in chemo-pretreated and chemo-naive patients would be interesting, we note that the numbers in each of these groups are small (ie, 17 and 37, respectively) and the results from such analysis have to be treated with extreme caution. Prospective, randomised trials would be necessary to address the question of the optimal timing of docetaxel administration, and therefore the best timing for phase-I consideration relative to conventional chemotherapy in CRPC.

Nonetheless, overall, these retrospective data provide preliminary clinical and ethical support for the exploration of novel agents both before and after docetaxel within early clinical trials, especially in CRPC patients where advances over the last two decades have been small.
